# Bronchial oncocytic carcinoma in an adult: a case report and literature review

**DOI:** 10.1186/s12890-023-02669-0

**Published:** 2023-10-06

**Authors:** Yi-Fan Shen, Cheng-Long Wang, Zhi-Gang Chu, You-De Cao, Yan Luo, Yang-Li Zhang, Yi-Jia Cao

**Affiliations:** 1 The Center for Clinical Molecular Medical Detection, The First Affiliated Hospital of Chongqing Medical University, Chongqing, China; 2 Department of Pathology, Chongqing Hospital of Traditional Chinese Medicine, Chongqing, China; 3 Department of Radiology, The First Affiliated Hospital of Chongqing Medical University, Chongqing, China; 4 Department of Pathology, College of Basic Medicine, Chongqing Medical University, Chongqing, China; 5 Department of Stomatology, the People’s Hospital of Dadukou District, Chongqing, China; 6 Department of General Surgery, the University-town Hospital of Chongqing Medical University, Chongqing, 401331 China

**Keywords:** Oncocytic carcinoma, Pulmonary, Prognosis, Case report

## Abstract

**Background:**

Lung salivary-type tumors originating from bronchial submucosal glands are rare, only four types of salivary gland-type tumors are listed in 2015 WHO classification of lung tumors. Here, we report a rare case of oncocytic carcinoma (OC) in the right main bronchus.

**Case presentation:**

A 34-year-old man presented to our hospital with a two-month history of recurrent hemoptysis and with one month of inspiratory dyspnea. Pulmonary function tests showed mild restrictive ventilatory dysfunction and severe diffusion dysfunction. Furthermore, the flow volume loop showed a variable extra-thoracic obstruction. Computed tomography (CT) of the chest revealed that a polypiform nodule of 13 mm in diameter was at the proximal right main bronchus. Testing for purified protein derivative was positive (category 2). The nodule was resected under bronchoscopy. The bronchial aspirate was negative for mycobacterium tuberculosis and tumor cells. The biopsy sample showed a solid and acinar predominant pattern with abundant eosinophilic cytoplasm. The bronchial mucosa was destroyed and replaced by tumor cells. The loose edematous stromal reaction could be seen in a local area. Immunohistochemically, tumor cells were positive for CK, EMA, Vimentin, CD117, CK7, S100, Mammaglobin and SOX10. Only scattered tumor cells were stained by basal cell markers, including CK5/6, P40 and P63. Electron microscopy revealed numerous swelling mitochondria with lacking mitochondrial cristae in tumor cells. Fluorescence in situ hybridization (FISH) testing for MAML2 and ETV6 rearrangement were negative. Next-generation sequencing analysis of 520 genes in the tissue biopsy specimen showed no somatic mutation. The diagnosis of OC was made. Subsequently, the patient underwent a right upper lobectomy with sleeve resection of the main bronchus and lymph dissection. No recurrent evidence was seen during two years of chest CT follow-up.

**Conclusions:**

To our knowledge, this is the first case of primary OC in the bronchus. This patient has no recurrence during two years of follow-up, indicating that primary OC in the bronchus has the same favorable prognosis as in salivary glands. Moreover, complete excision and thorough sampling to know the invasive growth pattern is important to reach the correct diagnosis.

**Supplementary Information:**

The online version contains supplementary material available at 10.1186/s12890-023-02669-0.

## Background

In bronchus, submucosal glands belong to salivary-type glands, so all types of salivary tumors theoretically can develop. But their occurrence is very low, so only four types of salivary gland-type tumors are listed in 2015 WHO classification of lung tumors, including mucoepidermoid carcinoma, adenoid cystic carcinoma, epithelial-myoepithelial carcinoma and pleomorphic adenoma. In recent years, some other salivary gland-type tumors have been found, such as hyalinizing clear cell carcinoma [1], mammary analogue secretory carcinoma [2] and myoepithelial carcinoma [3].

Oncocytes are epithelial cells characterized by numerous mitochondria, resulting in an eosinophilic granular cytoplasm. Salivary gland tumors formed by oncocytes can be classified as nodular oncocytic hyperplasia (oncocytosis), oncocytoma, and OC according to 2017 WHO classification of head and neck tumors. In the salivary gland, the lesions are rare and only account for approximately 1% of primary salivary gland tumors. To our knowledge, in the respiratory tract, oncocytosis and oncocytoma have been reported occasionally [4, 5], but OC has never. Here, we report a rare case of primary OC in the right main bronchus.

## Case presentation

**Fig. 1:**
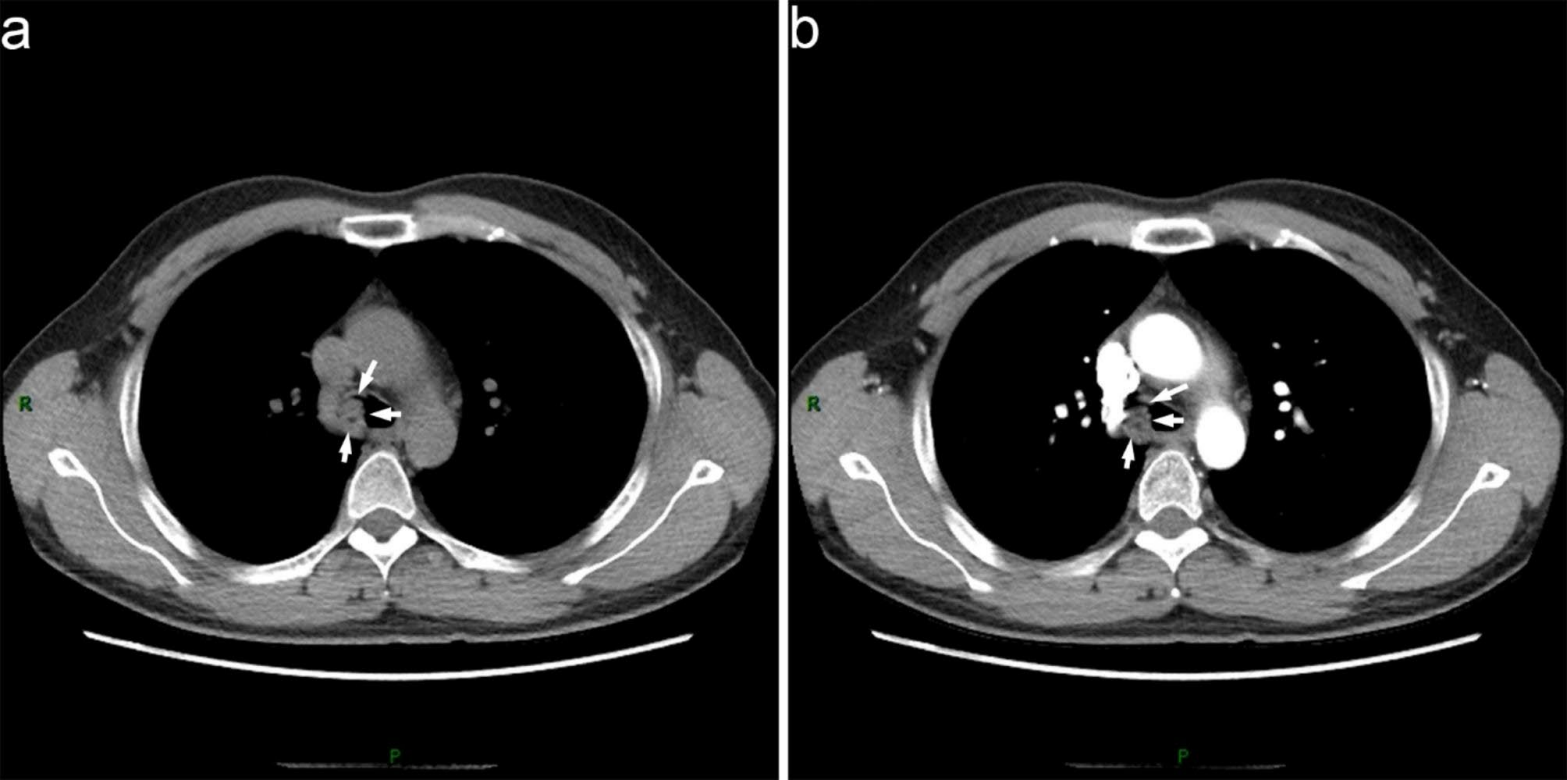
CT of chest showed the nodule. **(a)** The nodule (white arrows) on the membranous portion of the right main bronchus. **(b)** The nodule (white arrows) without enhancement on the enhanced chest CT.

**Fig. 2:**
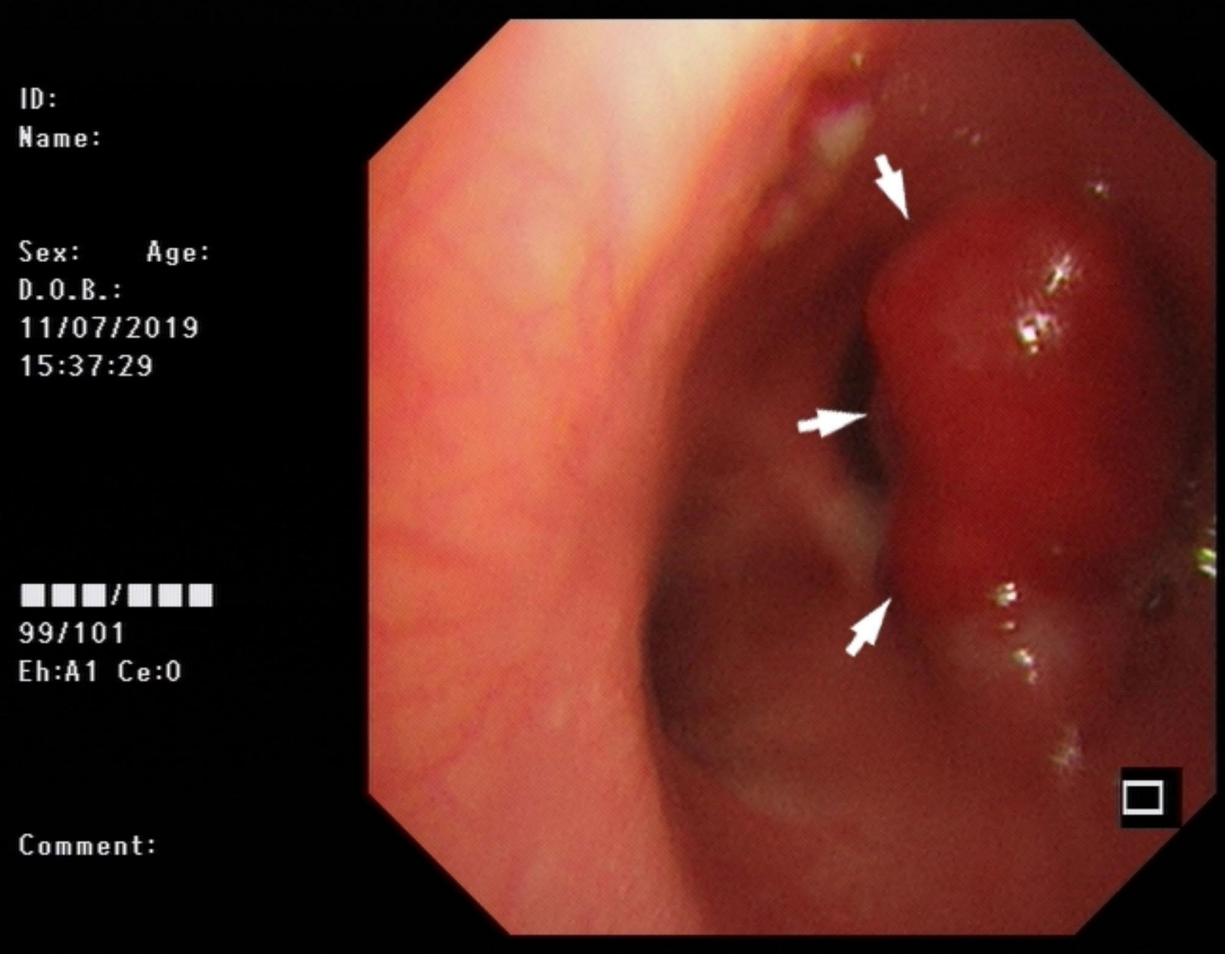
The bronchoscope showed the nodule. A red polypoid nodule (white arrows) occupied the proximal right main bronchus

A 34-year-old man presented to the respiratory clinic with a two-month history of recurrent hemoptysis (approximately 10ml/day) and with one month of inspiratory dyspnea. Pulmonary function tests showed mild obstructive ventilatory dysfunction (FEV1%F 66.47 and FEV1 92% of predicted values). Furthermore, the morphology of the flow volume loop showed a flattening of the expiratory limb, indicating a variable intra-thoracic obstruction. CT of the chest revealed that a polypiform nodule of 13 mm diameter was based on the proximal right main bronchus and about 2 mm above the carina, and protruded into the trachea with a broad-based connection to the wall (Fig. 1a), an additional movie file shows this in more detail [see Additional file 1]. Enhanced CT of the chest showed a non-enhancing nodule (Fig. 1b), an additional movie file shows this in more detail [see Additional file 2]. The purified protein derivative (PPD) test was positive (category 2). The bronchoscopic examination showed a red polypoid nodule occupied the proximal right main bronchus (Fig. 2). The nodule was resected by electrosurgical snare (SnareMaster 25 mm; Olympus Medical. Systems, Tokyo, Japan) 2 times using the power of 35 W. The resected sample was submitted to the pathology department for examination, and the diagnosis of OC was made. The bronchial aspirate was negative for mycobacterium tuberculosis and tumor cells. Subsequently, the patient underwent a right upper lobectomy with sleeve resection of the main bronchus, and dissection of the mediastinal, hilar and peribronchial lymph nodes at station 2 (n = 1), 4 (n = 5), 7 (n = 4), 9 (n = 1), 10 (n = 7), 11 (n = 5) and 12 (n = 2). The patient had no recurrent evidence during two years of chest CT follow-up.

**Fig. 3:**
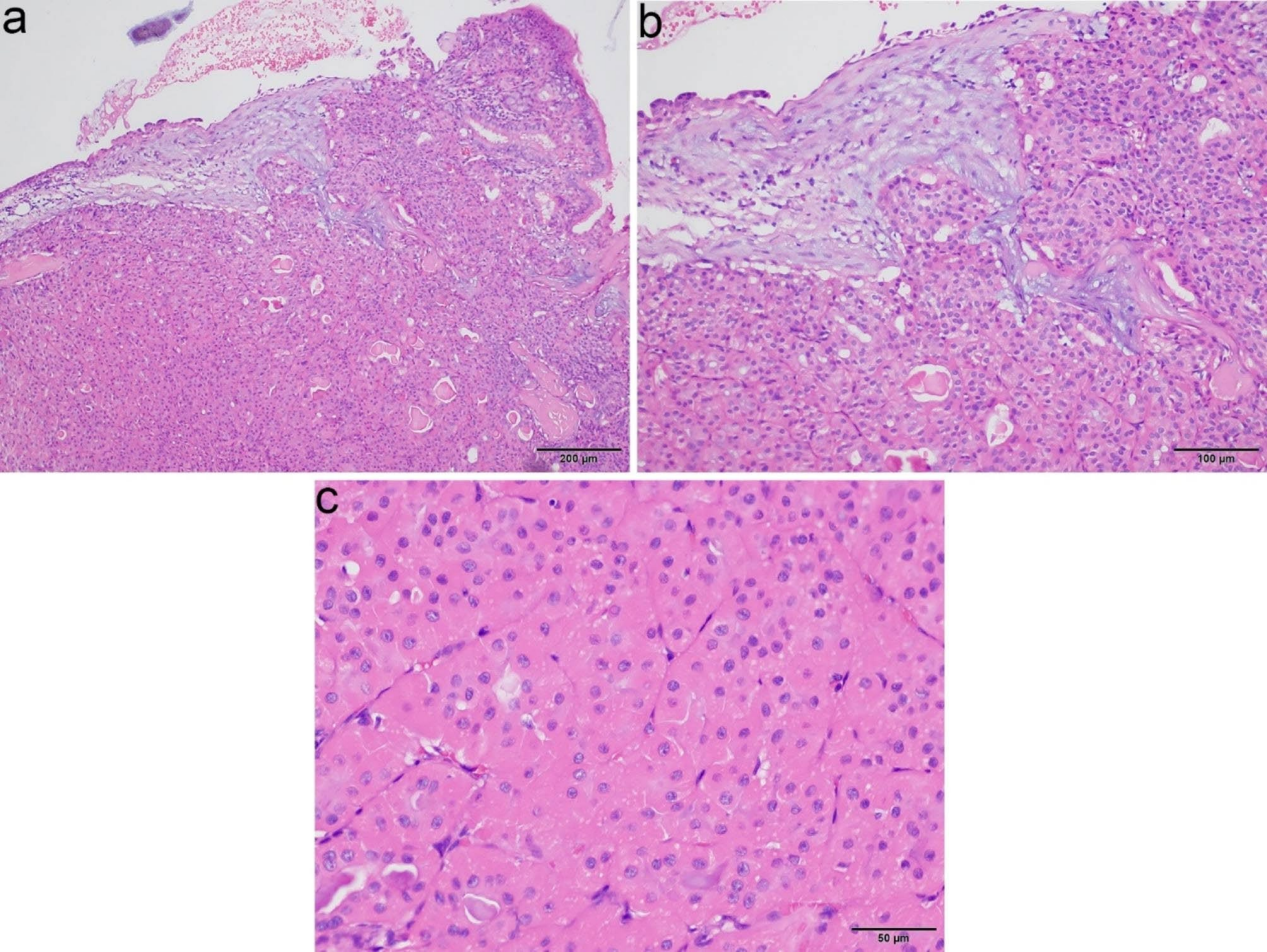
Morphology of the nodule. **(a)** The tumor invades and replaces the normal bronchial mucosa and submucosal glands with a focal stromal reaction and some acinar secretion. **(b)** High magnification of Fig. 3a, the loose edematous stromal reaction in the center is appreciated; the tumor cells are arranged in a nest and acinar pattern. **(c)** Oncocytic carcinoma shows abundant eosinophilic cytoplasm and centrally located vesicular nuclei with a single conspicuous nucleolus. Equipment used to obtain images: Olympus BX43F microscope, Olympus DP74 camera and acquisition software: OLYMPUS cellSens Dimension 1.16 software at a resolution of 1600 × 1200 pixel. The downstream processing to merge images in Adobe Photoshop CS6 at a resolution of 300 dpi

**Fig. 4:**
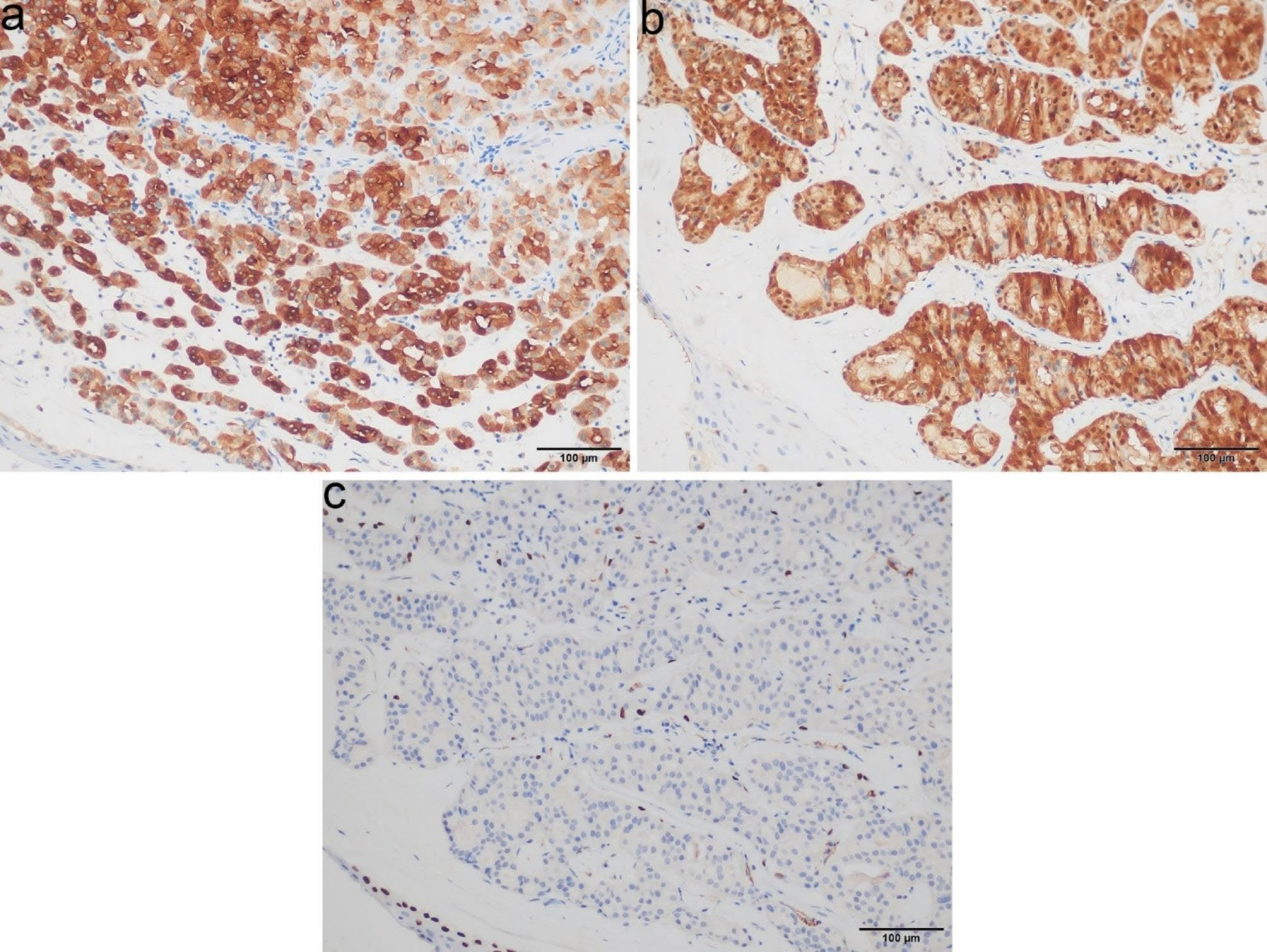
Immunohistochemical staining and ultrastructure of the nodule. **(a)** Mammaglobin shows diffuse cytoplasmic staining in tumor cells. **(b)** S100 shows diffuse cytoplasmic and nuclear staining in tumor cells. **(c)** P63 positive cells are present at the periphery of tumor nests. Equipment used to obtain images: Olympus BX43F microscope, Olympus DP74 camera and acquisition software: OLYMPUS cellSens Dimension 1.16 software at a resolution of 1600 × 1200 pixel. The downstream processing to merge images in Adobe Photoshop CS6 at a resolution of 300 dpi

The two submitted resected specimens measured 1.6 × 1.5 × 0.5 cm. On sectioning, the lesion was tan-white and firm. Histologically, the tumor cells were arranged in a nest and acinar predominant pattern. The bronchial mucosa was destroyed and replaced by tumor cells (Fig. 3a). The loose edematous stromal reaction was seen in a local area (Fig. 3b). PAS stain with amylase digestion showed that the acinar secretion was positive. Neurovascular invasion and obvious mitosis were absent. Cytologically, the bland cells had abundant eosinophilic granular or vacuolated cytoplasm with small red nucleoli, and intranuclear inclusions were seen in individual cells (Fig. 3c). Immunohistochemically, tumor cells were positive for CK, EMA, Vimentin, CD117, CK7, Mammaglobin (Fig. 4a), S100 (Fig. 4b) and SOX10, but negative for DOG1, CD56, Cga, Syn, CK20, Calponin, SMA, NapsinA and TTF1. Scattered basal cell marker stained in the tumor, including CK5/6, P40 and P63 (Fig. 4c). The majority of basal cell marker positive cells were present at the periphery of tumor nests. Small fragments obtained from paraffin blocks of the sample biopsy were used for the ultrastructural investigation, which showed that the tumor cells were engorged with numerous mitochondria. The mitochondria were enlarged and lacked lamellar cristae [see Additional file 3]. These structures were considered responsible for the eosinophilic cytoplasm of the tumor cells as seen by light microscopy. Fluorescence in situ hybridization (FISH) testing for MAML2 and ETV6 rearrangement were negative. To explore potentially actionable mutations, next-generation sequencing was performed on the tumor specimen using a panel consisting of 520 genes (OncoSreen Plus, Burning Rock Biotech, Guangzhou, China). No somatic mutation was detected from the genes included in the gene panel. The tumor cells were absent in the following resected bronchus, lung and lymph nodes.

## Discussion and conclusions

Oncocytosis, oncocytoma and OC form a spectrum of morphology and behavior. Their eosinophilic cytoplasm has been regarded as the accumulation of numerous mitochondria through electron microscopy, phosphotungstic acid hematoxylin and antimitochondrial staining [6, 7]. However, only in oncocytoma and OC, the swelling hyperplasic mitochondria are characterized by the loss of mitochondrial cristae [5], as is those of this case. Compared to oncocytoma, OC shows some relatively aggressive behaviors such as invading surrounding connective tissues, neurotropism and/or lymph-vascular invasion. In this case, tumor cells invaded and replaced the normal bronchial submucosal glands with focal stromal reaction, demonstrating the malignant behavior of OC.

To our knowledge, the primary OC in the respiratory tract has never been documented, and the oncocytosis and oncocytoma also are very rare with only seven cases documented (Table 1) [4–6, 8–11]. We found that 2 of 6 (33.33%) oncocytomas were mistaken as carcinoid tumor and mucoepidermoid carcinoma in the preoperative biopsy. OCs are difficult to be differentiated with other malignant and benign tumors in trachea and main bronchi on CT images, such as the squamous cell carcinoma, carcinoid carcinoma and hamartomas. In carcinoid tumor, calcification or marked enhancement may be seen at enhanced CT. While benign neoplasms including papillomas and hamartomas usually demonstrate as a well-defined, round and glossy lesion, and the polypoid configuration and intraluminal location of the mass can be seen on CT. Hamartomas and lipomyoma may be definitively diagnosed at CT if fat can be detected in them. The diagnosis of OC, the oncocytic variant of carcinoid tumor and that of mucoepidermoid carcinoma may be challenging due to the significant morphologic overlap between tumors. However, they have specific immunophenotypes. Having abundant neuroendocrine granules, carcinoid tumor is positive for neuroendocrine markers that are negative in OC. Although OC and the oncocytic variant of mucoepidermoid carcinoma have the same eosinophilic cytoplasm caused by numerous mitochondria accumulation, they have different origins. Mucoepidermoid carcinoma is thought of as the counterpart of the modified myoepithelial cells, so myoepithelial markers (e.g., P63, P40 and CK5/6) stain the majority of tumor cells [12]. In OC, a basal cell population is also present but its distribution pattern is characteristic. The majority of positive basal cells are present at the periphery of tumor nests, with some foci showing a diminishing gradient of staining towards the center of the nests. Moreover, MAML2 gene rearrangement may be helpful to support mucoepidermoid carcinoma.

**Table 1:** Summary of previous reports of oncocytosis, oncocytoma and OC cases in respiratory tract

Age/ Sex	Location	Biopsy diagnosis	Final diagnosis	Symptom	Identification of mitochondrion	Surgical method	Outcome	References
70/M	Trachea	N/A	Oa^a^	Hemoptysis	N/A	Endoscopic resection	Unknown	[8]
16/F	Trachea	LGMC	Oa	Hemoptysis	N/A	Lower tracheal resection	Survived	[9]
80/M	Bronchus	Os	Os	Unknown	N/A	N/A	Unknown	[4]
50/F	left lung	OA	Oa^b^	Dyspnea	PTAH, AT	N/A	Unknown	[10]
22/F	right lower lobe	N/A	Oa	Unknown	EM	Enucleation	Unknown	[5]
68/M	right upper lobe bronchus	CT	Oa	Malaise	PTAH, EM	Right upper lobectomy	Unknown	[6]
51/F	right middle lobe	Negative	Oa	None	EM	Right middle lobectomy	Survived	[11]
34/M	right bronchus	N/A	OC	Hemoptysis	EM	Right upper lobectomy with sleeve resection of the main bronchus	Survived	Present case

LGMC: low-grade mucoepidermoid carcinoma; PTAH: phosphotungstic acid hematoxylin; AT: antimitochondrial staining; OC: oncocytic carcinoma; Oa: oncocytoma; Os: oncocytosis; CT: carcinoid tumor; EM: electron microscopy; Unk: unknown; N/A: not applicable; ^a^ This oncocytoma with a concurrent pulmonary adenocarcinoma in left upper lung lope; ^b^ intrapulmonary multicentric oncocytoma;

Intriguing, this tumor is diffusely positive for S100 and Mammaglobin. The co-expression occurs in almost all secretory carcinoma, the majority of polymorphous adenocarcinoma and a minority of adenoid cystic carcinoma [13]. In this case, FISH was negative for ETV6 gene rearrangement characteristic of secretory carcinoma, and the staining pattern of basal cell markers did also not support the two basal/myoepithelial cell predominant tumors: polymorphous adenocarcinoma and adenoid cystic carcinoma.

OC in salivary glands is a low-grade malignant tumor, and complete excision is generally curative according to the latest WHO classification. The primary OC in the bronchus is also likely a low-grade malignant tumor with a favorable prognosis based on the two years follow-up results and the bland cytology. Simultaneously, the bland cytology in OC can be a diagnostic challenge in the forceps biopsy and cytological examination because it is difficult to observe the invasive growth pattern. Therefore, complete excision and thorough sampling to know the invasive growth pattern is important to reach the correct diagnosis. The electrosurgical snare is an alternative heat ablative therapy in the airway that uses an electrical current to generate heat and cause tissue destruction. And it is often used to lasso a lesion at its base, which is useful in pedunculated airway lesions [14]. We suggest that the electrocautery snare could be the first option to treat and diagnose a low-grade malignant broad-based airway lesion.

To our knowledge, this is the first case of primary OC in the bronchus. This patient has no recurrence during two years of follow-up, indicating that primary OC in the bronchus has the same favorable prognosis as in salivary glands. As its tricky bland morphology, complete excision and thorough sampling to know the invasive growth pattern is important to reach the correct diagnosis. Moreover, ultrastructural analysis of mitochondria, molecular genetic testing and immunohistochemistry are useful in facilitating the correct diagnosis.

### Electronic supplementary material

Below is the link to the electronic supplementary material.

Supplementary Material 1

Supplementary Material 2

Supplementary Material 3

## Data Availability

The raw NGS sequence data presented in this study can be found online in the National Center for Biotechnology Information repository (SRA: PRJNA935421), https://www.ncbi.nlm.nih.gov/Traces/study/?acc=%20PRJNA935421&o=acc_s%3Aa.

## Abbreviations

OC: Oncocytic carcinoma
CT: Computed tomography
FISH: Fluorescence in situ hybridization
