# Supplementary material for: Bronchial oncocytic carcinoma in an adult: a case report and literature review
Source: BMC Pulm Med. 2023 Oct 6;23:375. doi: 10.1186/s12890-023-02669-0 (PMC10559420; doi:10.1186/s12890-023-02669-0)
Supplement: Supplementary file 1 — Supplementary Material 1 [file 12890_2023_2669_MOESM1_ESM.docx]

**Additional file 1** Sequential CT images of chest showed the nodule. The nodule almost completely blocked the right main bronchus.

**Additional file 2** Sequential enhanced CT images of chest showed the nodule. The nodule showed no enhancement.

**Additional file 3** Ultrastructure of the nodule. **a.** Despite the artefact resulting from fixation in formalin, electron microscopy reveals numerous mitochondria with swelling in tumor cells. **b.** High magnification of the tumor cytoplasm ultrastructure, the swelling mitochondria lack mitochondrial cristae. Equipment used to obtain images: JEOL JEM-1400 Transmission Electron Microscope, Olympus Morada G2 camera and acquisition software: RADIUS 1.4 at a resolution of 3148 x 2096 pixel. The downstream processing to merge images in Adobe Photoshop CS6 at a resolution of 300 dpi.

| 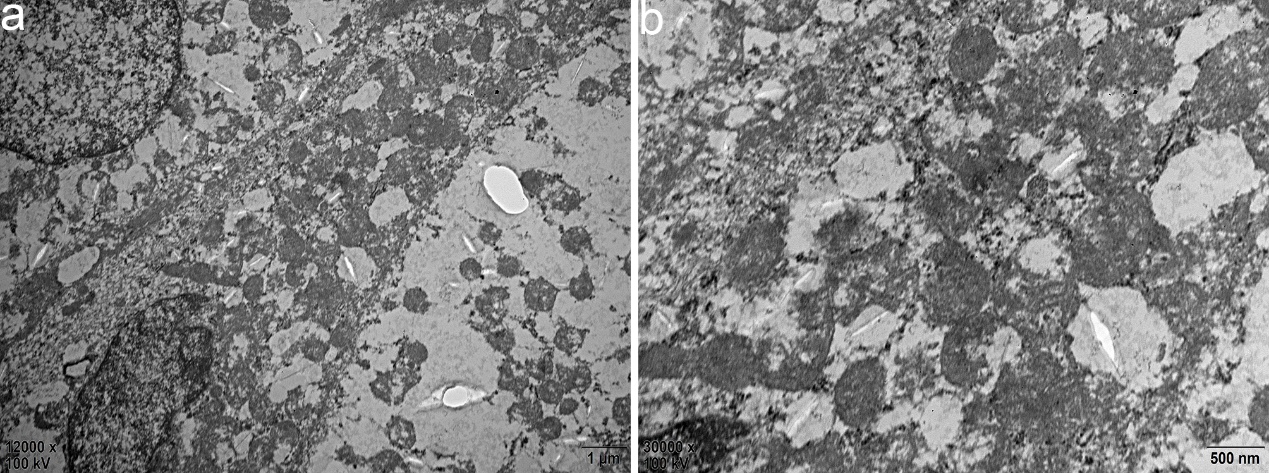 |
| --- |
